# Spectacles

**Published:** 1873-07

**Authors:** 


### SPECTACLES.

Let us commence this article by asserting that not one person in a hundred,—and the estimate is a safe one—knows *why* he requires the aid of lenses to assist vision, and what should be selected. It is also true that comparatively few so-called opticians know how to fit glasses to eyes that require them; so that frequently more real injury is done the eye in wearing spectacles than would have occurred had the patient attempted to read without them.

Before proceeding to give rules for the selection of spectacles, let us refer to some physiological facts or laws pertinent to the subject under consideration: Almost every one accustomed to using the eyes to gain a livlihood, has observed that when looking at a distant object, an appreciable moment of time elapses before the eye can adjust itself to see a minute object close at hand. To illustrate this fact, stand a short distance from a window and view the objects on the lawn or street; then, without moving your head, change your point of vision to the particles of dust or blemishes upon the window-glass. You will be conscious of an effort in the eye to accommodate itself to this new position. Now, gradually approach the glass, when you will find that the nearer you get your eyes to the glass, the greater will be the feeling of effort, until your face approaches so near the particles viewed that they become blurred and indistinct, when you involuntarily rub or press upon the eye-ball to relieve it of a tired, unpleasant feeling. A better experiment would be to place three pins upright on the table; one at eighteen inches from the eye, another at twenty-four, and the last at thirty. Then cast your eye along the table and fix your vision upon the head of the pin most distant. Those in front will appear blurred and indistinct. Look at the centre one, when the first and last will be out of focus, and therefore blurred. In this manner you will speedily discover that the eye requires the same adjustment for near and far objects that you give to your opera glass. In viewing objects at varying distances through the opera-glass, you find it necessary to adjust it with the screw in order to see them plainly. In observing the same objects with the naked eye, a similar adjustment takes place, and is accomplished by the *ciliary muscle*, which renders the lens of the eye more or less convex, to correspond to the distance of the object observed. This involuntary adjustment of the eye is termed *accommodation*, and is a muscular effort; therefore, when we compel the eye to view near objects for a considerable time, as in reading, sewing, or any employment where close and constant vision is necessary, we subject it to the general laws which govern muscular activity, and it becomes tired. It is understood then, that the muscular effort necessary to adjust our eyes is accompanied with fatigue, and when persisted in, with pain, when the objects become blurred, the lines in reading become indistinct, and run together, tears flow freely, and we are admonished to cease our labors and give our visual organs the required rest.—This power of adjustment is found to be greatest in youth, when the lens is soft, and therefore easily rendered more or less convex by the action of the ciliary muscle. As we advance in years the lens becomes harder, and less yielding, so that more effort is necessary for adjustment, until between 40 and 45 years of age, when it becomes almost impossible for the muscle to act upon the lense, so hard and unyielding has it become. We therefore witness our friends of this age gradually holding the paper farther from them, turning their backs to the light so that they may secure better illumination of the page, contracting their eye brows, and finally throwing the paper down with an exclamation that their eyes have given out. This is the proper moment for the adjustment of suitable spectacles; but, as the idea of approaching old age is associated with the wearing of glasses, and as none of us like to confess that we are growing old, we are apt to neglect this admonition of our visual organs and tax them beyond their endurance, until absolute and irremediable harm is done them, compelling the persons so foolish as to rather be considered young than healthful, to plod along through the balance of their lives with crippled and imperfect vision.

The wearing of spectacles is *not* an evidence of old age. Many eyes have lost their power of accommodation in childhood, when the action of the lens and ciliary muscle are at their height of capability for quick adjustment. We are frequently, yes, almost daily called upon to adjust glasses to children of from 5 to 12 years of age, that would have been suitable for people of 80 years. This defect in children's eyes is known to ophthalmic surgeons as *hypermetropia* or "over-sight," in which condition the eye is so elongated that the power of accommodation is not strong enough to render the lens sufficiently convex to bring the image of objects observed upon the retina.

It should therefore be understood that the wearing of glasses is by no means an evidence of old age, for at the age of 40 to 45 is the very prime of man's vigor, yet, is it the usual time for adopting the use of glasses to assist the eye in that adjustment which has become enfeebled through the constant use which, in these busy days, business men are constantly subjecting them to. For, be it remembered, our eyes are constantly in use,—save during the hours of sleep, while the rest of the body is at ease, therefore it is not surprising that some time they will *demand* the rest which we have continually refused them.

We often hear people of 50 years of age, or even more, boasting they have never required the use of glasses. Watch them:—they select only books or newspapers of the plainest print, seat themselves in the brightest light, and hold the paper well away from the eye. They are careful not to do any reading by nightfall, and as soon as the eye becomes fatigued they have the good sense to desist from reading. Some old people, from having a very small pupil, by reason of which the marginal rays of light are cut off from the eye, rendering the central ones nearly parallel, are able to see quite clearly without glasses. Others, having worn glasses, are enabled to throw them off, seeing better without them, and rejoice that their sight is being "renewed." Such rejoicings are usually cut short in ultimate dimness of vision, and finally, blindness; the temporarily improved vision being the result of a swollen lens, previous to its taking on a *cataractous* form, eventuating in total blindness.

Then, there is another morbid condition of the eye which requires stronger and stronger glasses. A person commences the use of spectacles at the usual time, but is compelled to constantly change them for stronger ones, finally being able to see only from the strongest magnifying glasses. Soon his eyes become painful, feel hard and tense to the touch, the field of vision becomes contracted, and objects appear to have a sort of halo about them. This is the approach of a very dangerous disease, known as *glaucoma*, and which results in incurable blindness, unless speedily arrested by the skilful ophthalmic surgeon.

Many people congratulate themselves upon being able to put off the wearing of glasses to later years than those of their acquaintances, thinking that they are strengthening their eyes thereby. This is a fatal error, as they compel their eyes to perform hard labor, which would have been comparatively easy under the use of suitable glasses, and would have preserved strong powers of vision to a much later period of life.

We have found that persons who have arrived at the age of 40 or 45, require aids to vision in the form of spectacles. How shall they select them? Emphatically not by going to the clock and jewelry dealer, and there "trying on" such glasses as may be handed them. Such dealers know nothing of the laws of accommodation or refraction, neither is it their business to know. They can tell you *about* the required strength of glass that would be suitable for a person of your age, and will sell you spectacles that *perhaps* will improve your vision, but it is much the safest plan to consult an oculist, who will test your vision and will perscribe the form and focal length of the lens suited to the condition of your eye. If your eyes are sound and healthy, lacking only in power of accommodation, the following table can be used as a guide for the selection of lenses, when no oculist is near for consultation. Glasses are numbered by their focal length in inches, the following are the usual numbers required:

| Age. | Convex Glasses required. | Distance of Distinct Vision in inches. |
|---|---|---|
| 40 } 45 } | 60 | 14 |
| 50 | 40 | 14 |
| 55 | 50 | 14 |
| 58 | 22 | 13 |
| 60 | 18 | 13 |
| 62 | 14 | 13 |
| 65 | 13 | 12 |
| 70 | 10 | 10 |
| 75 | 9 | 9 |
| 78 | 8 | 8 |
| 80 | 7 | 7 |

A variety of circumstances and conditions of the eye are taken into consideration by the ophthalmic surgeon in selecting glasses for his patient, that cannot be explained in an article like this. For instance, whether the glasses are to be selected are for use in reading proof, manuscript, music, or in pulpit reading. Whether they are to be employed in engraving, watch-repairing, sewing, drawing, type setting, or whether to be in use night or day or both. Whether any abnormal condition of the eye exists, as astigmatism, paralysis of accommodation, insufficiency of any of the muscles, etc., etc.; then, he is to decide what form of glass is to be employed, whether round or oval, and how large, and whether perfectly transparent or tinted lenses should be used. These are very important points to be decided, and upon their proper decision much of the comfort and happiness of the patient depends. If you are compelled to select your glasses without professional aid, your best plan is to go to some reliable dealer, who is intelligent and honorable enough to deal in nothing but accurately ground lenses, made of good quality of glass, or what is better, of pebble, and then select a number corresponding to your age, as given in the above table. If the spectacles enable you to read ordinary print, at the required distance, without magnifying the type or causing brilliant colors to appear about them; and, after using them a half hour, your eyes do not feel weary but refreshed from their use, you would be tolerably safe in purchasing and using such a pair. But, if you have to select glasses five or six numbers stronger than indicated in our table, to enable you to read, it would be an evidence of some abnormal condition of your eye, and the wearing of them would be extremely dangerous, without the consent or advice of an oculist. As before remarked, lenses made of pebbles are much the best. They are clearer, harder, and less easily marred than any other. It is important that lenses should be accurately ground, and for this reason those of reliable makers should be selected. If the eyes are inclined to be "weak," or you are compelled to wear glasses in the sun or gas light, violet-tinted lenses would be much more comfortable, as they exclude the heat-rays of light and do not perceptibly diminish the light-giving rays. Such lenses are now manufactured, and can be obtained of Mr. H. W. Strang of this city, or any extensive dealer in optical goods.

We have dwelt so long upon the selection of glasses for old people, or for those who are becoming *presbyopic*, or "old sighted," that we find no room left us in which to treat of near-sighted glasses, and their proper selection. We must therefore leave this subject for some future issue of the Bistoury. Many abuses to which "old eyes," are subjected, have been deprecated in former articles upon this subject, such as rubbing the eyes or using "eye-cups," or "eye sharpeners," with the intention of elongating the eye, and so trying to do away with glasses. Such practices are extremely hazardous, and worse than useless. When the period arrives rendering it necessary to seek assistance to the visual powers, it is a grave error to persist in endeavors to do away with the use of glasses. They serve to prevent straining the eyes and preserve good vision to a good old age. Therefore, when you find the need of them, use them, selecting them carefully and under advice, allowing no false sense of pride to debar you from employing means that will afford you lasting comfort, ease and pleasure.

